# State of the Art in CAR-T Cell Therapy for Solid Tumors: Is There a Sweeter Future?

**DOI:** 10.3390/cells13090725

**Published:** 2024-04-23

**Authors:** Beatriz Amorós-Pérez, Benigno Rivas-Pardo, Manuel Gómez del Moral, José Luis Subiza, Eduardo Martínez-Naves

**Affiliations:** 1 Department of Immunology, Ophthalmology and ORL, School of Medicine, Universidad Complutense of Madrid (UCM), 28040 Madrid, Spain; beamoros@ucm.es (B.A.-P.); benignor@ucm.es (B.R.-P.); 2 Instituto de Investigación Sanitaria Hospital 12 de Octubre (imas12), 28041 Madrid, Spain; 3 Inmunotek S.L., 28805 Madrid, Spain; jlsubiza@inmunotek.com; 4 Department of Cellular Biology, School of Medicine, Universidad Complutense of Madrid (UCM), 28040 Madrid, Spain; mgomezm@med.ucm.es

**Keywords:** cancer, immunotherapy, chimeric antigen receptors, CAR-T cells, solid tumors

## Abstract

Chimeric antigen receptor (CAR)-T cell therapy has proven to be a powerful treatment for hematological malignancies. The situation is very different in the case of solid tumors, for which no CAR-T-based therapy has yet been approved. There are many factors contributing to the absence of response in solid tumors to CAR-T cells, such as the immunosuppressive tumor microenvironment (TME), T cell exhaustion, or the lack of suitable antigen targets, which should have a stable and specific expression on tumor cells. Strategies being developed to improve CAR-T-based therapy for solid tumors include the use of new-generation CARs such as TRUCKs or bi-specific CARs, the combination of CAR therapy with chemo- or radiotherapy, the use of checkpoint inhibitors, and the use of oncolytic viruses. Furthermore, despite the scarcity of targets, a growing number of phase I/II clinical trials are exploring new solid-tumor-associated antigens. Most of these antigens are of a protein nature; however, there is a clear potential in identifying carbohydrate-type antigens associated with tumors, or carbohydrate and proteoglycan antigens that emerge because of aberrant glycosylations occurring in the context of tumor transformation.

## 1. Introduction

Cancer immunotherapy has become the forefront of modern oncology, representing one of the most effective strategies to treat diverse human malignancies. Unlike traditional treatments that directly target cancer cells, immunotherapy harnesses the intricate machinery of the immune system to combat the disease. By leveraging the innate capability of the body to identify and eliminate abnormal cells, including those implicated in cancer, immunotherapeutic interventions offer the promise of durable responses and even complete remissions. Immune checkpoint inhibitors are the most successful immunotherapeutic treatments to date.

Despite this, the overall response rate is still unsatisfying. Therefore, new approaches are being developed [1]. Among the arsenal of immunotherapeutic approaches under development, genetically engineered T cell therapies, exemplified by chimeric antigen receptor T cells (CAR-T) have emerged as a beacon of hope in the realm of cancer management.

**Figure 1:**
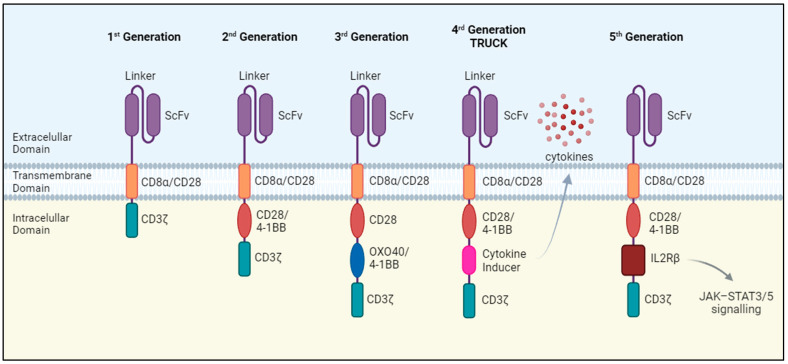
Generations of CAR-T cell therapy. The different generations have been built adding co-stimulatory domains to the first-generation structure. Second- and third-generation CAR-T cells usually include a CD28 and/or a 4-1BB domain. Fourth-generation CAR-T cells (or TRUCKs) contain a domain to release a transgenic cytokine upon CAR signaling. Fifth-generation CARs add a truncated cytoplasmic IL-2 receptor β-chain to the second-generation structure.

Chimeric antigen receptors (CARs) transduce antigen-recognition events into a signaling cascade that triggers T cell effector functions, including the release of cytotoxic factors and pro-inflammatory cytokines. The essential components of these synthetic receptors include an extracellular antigen-recognition domain, a hinge or spacer region, a transmembrane domain that anchors the receptor on the cell surface, and an intracellular signaling domain. The antigen-recognition domain is a single-chain variable fragment (scFv) consisting of the variable regions of the immunoglobulin heavy and light chains connected by a peptide linker which recognizes tumor surface antigens. Upon recognition, CAR-T cells are activated through signals transmitted by the intracellular domain, whose structure and functionality depends on the generation of the CAR-T cell (Figure 1).

The first generation of CAR-T cells consisted of the scFv domain joined with a single intracellular domain, most frequently the T cell receptor (TCR) CD3ζ signaling chain. These CAR-T cells showed a weak expansion potential in vivo and no antitumor effect [2]. Therefore, the second generation of CAR-T cells emerged, incorporating an extra intracellular motif: the signaling domain of co-stimulatory receptors such as CD28 and 4-1BB. These domains provide additional activation signals, resulting in enhanced antitumoral efficacy and improved survival and persistence of CAR-T cells in vivo [3,4] CAR-T cell therapies approved to date belong to this generation.

Despite the success achieved by the second generation of CAR-T cells, not all patients exhibit a positive response to this therapy [5]. To overcome this limitation, a third generation of CAR-T cells was developed that incorporated two costimulatory domains, leading to enhanced proliferation, activation, and antitumor efficacy [6].

The fourth generation of CAR-T cells takes a step further by introducing additional modifications, such as a constitutive or inducible expression of chemokines. T cells redirected for universal cytokine-mediated killing (TRUCKs) are designed to express and release various types of cytokines such as interleukin-12 (IL-12), IL-15, or IL-18, thereby improving T cell activation and antitumor efficacy [7,8,9]. Cytokine release has the potential to induce a proinflammatory milieu within solid tumors and influence the tumor microenvironment [10]. Fifth-generation CAR-T cells follow the structure of the second generation but with the addition of a truncated cytoplasmic IL-2 receptor β-chain (IL-2Rβ). Antigen recognition triggers the activation of TCR signaling through the CD3ζ domain, co-stimulatory signaling via CD28 and the JAK/STAT pathway due to the IL-2Rβ, which are the three synergistic physiological pathways essential for full T-cell activation [11].

## 2. Approved CAR-T Cell Therapies

CAR-T therapies have emerged as revolutionary treatments for various hematological cancers, with notable success in targeting CD19 and B-cell maturation antigen (BCMA). CD19 is expressed on differentiated B-cell lineages as well as malignant B-cells. BCMA is an antigen found at high levels on the surface of plasma cells in patients with multiple myeloma (MM), making it an ideal target for treating this disease.

In the realm of CD19-targeted CAR-T cells, four therapies have received approval by the Food and Drug Administration (FDA) and the European Medicines Agency (EMA): axicabtagene ciloleucel (axi-cel), tisagenlecleucel (tisa-cel), lisocabtagene maraleucel (liso-cel), and brexucacabtagene autoleucel (brexu-cel). Axi-cel was approved by the FDA in 2017 for the treatment of adult patients with relapsed or refractory (r/r) large B-cell lymphoma after two or more prior lines of therapy, including diffuse large B-cell lymphoma (DLBCL) not otherwise specified (NOS), primary mediastinal large B-cell lymphoma (PMBCL) and high-grade B-cell lymphoma (HGBCL). This marked the first approval of a CAR-T cell therapy [12]. Tisa-cel received approval later for the treatment of r/r B-cell acute lymphoblastic leukemia (ALL) in children and young adults, and for the treatment of adults with DLBCL and follicular lymphoma (FL) [13,14]. Liso-cel was the third major CD19 CAR-T approved. It is used to treat adult patients with r/r DLBCL, PMBCL, HGBCL, and FL grade 3b [15]. Brexu-cel was the first CD19 CAR-T approved for the treatment of r/r mantle cell lymphoma [16], a B cell lymphoma with extremely poor prognosis when chemoimmunotherapy and Bruton’s tyrosine kinase (BTK) inhibitors are ineffective [17].

In the case of BCMA-targeted CAR-T therapies, idecabtagene vicleucel (ide-cel) and ciltacabtagene autoleucel (cilta-cel) have received approval for the treatment of adults with r/r MM after receiving three or more previous therapies, including an immunomodulatory agent, an anti-CD38 antibody, and a proteasome inhibitor [18,19].

The treatments approved to date with CAR-Ts have in common that they are limited to hematological cancer and that the antigens they target are expressed in both tumor cells and healthy cells, that may be dispensable.

## 3. Challenges and Limitations of CAR-T Cell Therapy in Solid Tumors

**Figure 2:**
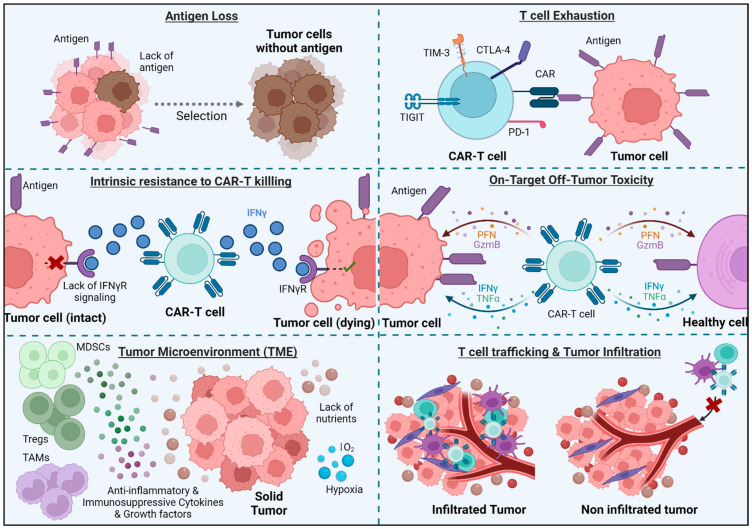
Limitations of CAR-T cell therapy. Some tumor cells lose antigen expression or have a defect in IFNγR signaling, which renders it difficult to evaluate CAR-T cell performance. Constant antigen exposure can also cause T cell exhaustion, and if the antigen is present on healthy cells as well, CAR-T cells can kill these non-tumor cells. Additionally, when treating solid tumors, CAR-T cells must face obstacles such as infiltration and remain active in the hostile TME.

The biology of solid tumors poses a major challenge for CAR-T-based therapies. While CAR-T cells have certain limitations that need to be addressed (Figure 2), these limitations may be more evident in the context of solid tumors.

### 3.1. Antigen Loss

Loss of antigen expression limits CAR-T efficacy on both solid and hematological tumors. In fact, one of the major causes of relapse in patients treated with CD19-CAR-T is antigen loss, occurring in 30–70% of patients who experience recurrent disease after treatment [20]. In this particular case, this can be produced by mutations and splice variants of the gene encoding the antigen [21], which causes that the malignant cells of the patients treated with CAR-T cell therapy display either a partial or complete loss of expression of the target antigen [20].

There are some strategies that can make CAR-T cell therapy more effective when there is a downregulation of the antigen, such as dual or bispecific CARs, which are CAR-T cells targeting two different antigens present on tumor cells. A phase I clinical trial with patients with r/r non-Hodgkin lymphoma (NHL) showed that autologous CD19/CD20 bispecific CAR-T cell therapy was safe and demonstrated strong efficacy (90% ORR, 70% CR rate) [22].

### 3.2. T Cell Exhaustion

The persistent exposure of T cells to their specific antigens causes their differentiation into an effector and dysfunctional phenotype, characterized by diminished proliferative capacity and function [23]. This differentiation is associated with poor responses to immunotherapy in cancer patients and the upregulated expression of “exhaustion” cell markers such as PD-1, TIM-3, LAG-3, TIGIT, and CTLA-4. In addition, it is associated with poor responses to immunotherapy in cancer patients [24]. Moreover, antibody blockade of these immune checkpoints, such as PD1/PD-L1, in combination with CAR-T cell therapy, has shown improved performance [25,26]. There are ongoing clinical trials of CAR-T cell therapy against solid tumors in combination with PD1/PDL1 (NCT05373147, NCT06249256, NCT05812326, NCT05631899, NCT05631886, NCT05089266) and CTLA-4 blockade (NCT06248697).

Other novel approaches are aimed at decreasing CAR–antigen interaction, as constant CAR stimulation by the antigen and exposure to TME causes T cell exhaustion. Weber et al. developed a drug-regulatable CAR-T cell incorporating an FK506 binding protein 12 (FKBP) destabilizing domain into a GD2-targeting CAR (GD2.28ζ.FKBP) and demonstrated that these modified CAR-T cells showed lower expression of inhibitory receptors PD-1, LAG-3, and TIM-3 than anti-GD2 CAR-T cells with sustained CAR expression [23]. Apart from adjusting CAR expression levels to regulate CAR signaling, the antigen-recognition component of CAR can be separated from the activation domain to address prolonged antigen stimulation in CAR-T cells. Switchable CAR constructs employ a soluble antigen-recognition domain, referred to as a switch, to target tumor-specific antigens. Administering the switch, which binds to the CAR portion on the cell surface to initiate signaling, enables reversible control of CAR activation in T cells, potentially preserving their effector function during periods of rest. Moreover, switch designs facilitate the substitution of CAR specificity as needed. Similarly, split CARs offer a mechanism to manage overactivation in cells by separating CAR activation from antigen recognition or co-stimulation. Split CAR-T cells targeting GP3 were effective against hepatocellular carcinoma cell lines in vitro and in vivo, but these split CAR-T cells produced less pro-inflammatory cytokines than conventional CAR-T cells [27].

### 3.3. T Cell Trafficking and Tumor Infiltration

CAR-T cell therapy in solid tumors is constrained by the ability of T cells to infiltrate the tumor mass and overcome physical tumor barriers, such as the tumor stroma, which limits the mobility and penetration of CAR-T cells [28]. There are several ways to improve the infiltration of CAR-T cells into the tumor:

#### 3.3.1. Local Administration

This method not only facilitates CAR-T cells’ access to the tumor environment but also mitigates off-tumor toxicities by reducing CAR-T cells’ interactions with normal tissue. Mayor et al. and Adusumilli et al. demonstrated that intrapleural injection of anti-mesothelin (MSLN) CD28 costimulatory (M28z) CAR-T cells effectively eliminated established pleural tumors, even at doses 30 times lower than those required for intravenous injection. Furthermore, locally injected CAR-T cells induced systemic and long-lasting antitumor immunity, with the ability to migrate easily from the thoracic cavity to adjacent flanking and peritoneal tumor sites. T cell imaging revealed that local administration resulted in earlier and increased accumulation of CAR-T cells within the tumor [29,30]. In a model of breast cancer metastasis to the brain, Priceman et al. compared regional intracerebroventricular (i.c.v.) to local intracranial (i.c.) HER2-CART cell delivery in BBM1 tumor-bearing mice. Equivalent antitumor responses and extended survival of mice after i.c. and i.c.v. delivery of HER2-BBζ CAR-T cells were observed. Notably, i.c.v. delivery of HER2-CAR-T cells exhibited delayed responses in some mice compared with local intratumoral delivery, likely owing to the required trafficking of these cells from the ventricle to the tumor site. Intraventricularly delivered HER2-BBζ CAR-T cell–mediated antitumor activity was also observed with larger tumor burdens [31].

#### 3.3.2. Expression of Chemokine Receptors

Chemokines play an essential role in homing and guiding the migration of immune cells [32]. Tumor cells secrete chemokines and cytokines that can attract pro-inflammatory or anti-inflammatory immune cells to the tumor microenvironment. Circulating IL-8 levels correlate with poor prognosis in multiple solid tumors, including melanoma, renal cell carcinoma, non-small cell lung cancer (NSCLC), and pancreatic, breast, and ovarian cancer [33]. CXCR2-expressing CAR-T cells against the integrin αvβ6 can migrate more efficiently towards tumor-conditioned media containing IL-8 and exhibit superior antitumor activity against established αvβ6-expressing ovarian or pancreatic tumor xenografts, with a more favorable toxicity profile [34].

#### 3.3.3. CAR-T Cells Engineered to Better Penetrate the Tumor Stroma

The extracellular matrix (ECM) is composed of various structural molecules such as fibrous proteins, proteoglycans, and glycosaminoglycans. These are produced by tumors and cancer-associated fibroblasts (CAFs). Fibroblast activation protein (FAP) α is a protease expressed by CAFs whose expression is higher on the surface of breast, lung, colorectal, prostate, stomach, pancreatic, prostate, thyroid, cervical, and urothelial cancers, and it is associated with poor prognosis [35]. FAP-directed CAR-T cells have been developed to target CAFs in several solid tumors, including mesothelioma, lung cancer, and pancreatic cancer, demonstrating antitumor activity in preclinical models [36,37,38]. A fourth-generation CAR-T targeting Nectin4/FAP has been developed against Nectin4 positive advanced solid malignancies, and it is being used in a phase I clinical trial (NCT03932565).

Another approach is to target the proteins present in the ECM. Hyaluronidase and α-PDL1-engineered CAR-T cells showed therapeutic efficacy on two solid tumor models and did not cause significant systemic side effects. The modified hyaluronidase degrades hyaluronic acid and destroys the tumor ECM, allowing CAR-T cells to penetrate deeply into solid tumors [39].

### 3.4. Immunosuppressive Tumor Microenvironment (TME)

The TME is often hostile to the activation and function of CAR-T cells, characterized not only by a limited supply of nutrients and oxygen due to insufficient blood flow [40], but also by the creation of an immunosuppressive environment. This suppressive environment is generated by tumor cells and other immune cells such as myeloid-derived suppressor cells (MDSCs), tumor-associated macrophages (TAMs), and regulatory T cells (Tregs). These cells produce cytokines, chemokines, and growth factors that promote tumor growth and cause T cell inhibition [41]. Generating CAR-T cells insensitive to some of these suppressing cytokines can help T cells overcome the TME. For example, transforming growth factor β (TGFβ) is one of the most important regulators in the TME, suppressing the cytotoxic function of CD8 T cells and promoting the generation of Tregs. Moreover, inhibition of TGFβ-signaling enhances tumor elimination by T cells. CAR-T cells insensitive to TGFβ (by knocking out TGFBR2 receptor using the CRISPR/Cas9 system) have shown improved effects in vitro and in PDX models [42]. A phase I clinical trial of castration-resistant prostate cancer has shown that TGF-β-resistant CAR-T cell therapy is feasible and generally safe [43]. Additionally, prostaglandins, particularly prostaglandin E2 (PGE2), play a significant role in the TME by modulating immune responses, often leading to the suppression of effective antitumor immunity. Their suppressive effect on CAR-T cell function within the TME is an area of active research, given their impact on various aspects of the immune response [44].

Third- or fourth-generation CAR-T cells can be utilized to overcome the immunosuppressive environment. IL-7- and CCL19-secreting CAR-T cells (7 × 19 CAR-T) demonstrated enhanced expansion and migration capabilities in vitro, along with superior tumor-suppression abilities compared to conventional CAR-T cells in xenografts of hepatocellular carcinoma (HCC) cell lines, primary HCC tissue samples and pancreatic carcinoma (PC) cell lines. A phase I clinical trial was conducted on advanced HCC/PC/ovarian carcinoma patients with glypican-3 (GPC3) or MSLN expression (NCT03198546). In one patient with advanced HCC, treatment using anti-GPC3-7 × 19 CAR-T resulted in the complete disappearance of the tumor 30 days post-intratumor injection. In another patient with advanced PC, treatment with anti-MSLN-7 × 19 CAR-T via intravenous infusion resulted in near-complete tumor disappearance after 240 days [45].

### 3.5. Intrinsic Resistance to CAR-T Killing

Tumor cells may have intrinsic resistance mechanisms to CAR-T-mediated cytotoxicity that differentiate them from solid or hematological tumors. In a recent study, Larson et al. [46] showed that the IFNγR signaling pathway is essential for CAR-T cells to kill tumor cells derived from solid, but not liquid, tumors. There is a need to better understand the killing mechanisms of CAR-T cells to optimize treatments depending on the type of tumor. To improve the outcome of CAR-T cell therapy in solid tumors, one approach could be to use TRUCKs that release other inflammatory cytokines to compensate for the lack of response to IFN-γ.

### 3.6. Combination Strategies to Improve CAR-T Therapies

**Figure 3:**
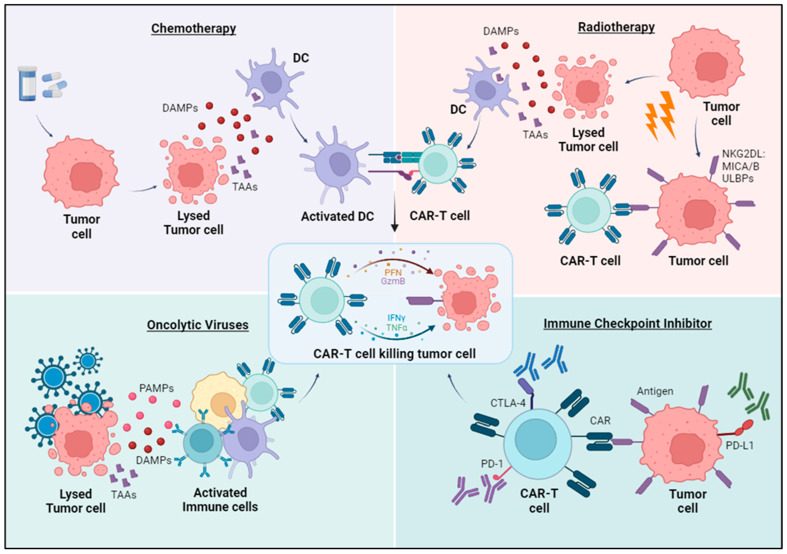
Combination of CAR-T cells with other antitumor therapies. Chemotherapy, radiotherapy, and oncolytic virus treatment can kill tumor cells and release danger-associated molecular patterns (DAMPs) and tumor-associated antigens (TAAs). These mechanisms promote the activation of innate and adaptative immune cells and potentiate the activity of CAR-T cells. Radiotherapy can also upregulate some antigens on tumor cells, such as NKG2D ligands, which can be targeted by CAR-T cells. The use of immune checkpoint inhibitors, as anti-PD1, CTLA-4, or PD-L1 antibodies, can decrease CAR-T cell exhaustion, thus contributing to a increasingly effective tumor killing by CAR-T cells.

Combining CAR-T cell treatment with other established cancer therapies is being tested as an alternative to monotherapy (Figure 3):

#### 3.6.1. Chemotherapy

When administered at low doses, chemotherapy plays an immunomodulatory role by stimulating dendritic cells’ activation and facilitating tumor antigen presentation to CAR-T cells. It also inhibits autoimmunity, and eliminates suppressive immunity, thereby enhancing the persistence of CAR-T. Additionally, it sensitizes tumor cells to CAR-T cell activity by promoting granzyme B penetration into tumor cells [47]. A preclinical study showed that administration of oxaliplatin and cyclophosphamide enhances chemokine expression and accumulation of CAR-T cells in a mouse model of ROR1+ lung cancer [48].

#### 3.6.2. Radiotherapy

Radiotherapy can regulate the ECM by increasing its permeability, thereby facilitating the infiltration of T cells [49]. Moreover, this therapy can modulate expression levels of tumor antigens used as targets for CAR-T cell treatment, such as NKG2D ligands, that are induced by ionizing radiation [50], and it potentially could be used in combination with NKG2D CAR-T cells for the treatment of triple-negative breast cancer [51]. Fractionated irradiation also upregulates expression of the immune checkpoint B7-H3 (CD276) on prostate cancer stem cells, and B7-H3 CAR T cells have demonstrated a potent cytotoxicity in vitro and significantly improved antitumor efficacy in mice [52]. In glioblastoma, a subtherapeutic dose of local radiotherapy combined with KG2D-based CAR-T cell treatment exhibited synergistic activity in two independent syngeneic mouse glioma models by promoting migration of CAR-T cells to the tumor site and increasing effector functions [53].

#### 3.6.3. Oncolytic Viruses

Oncolytic viruses exhibit the ability to infect and selectively replicate within tumor cells, causing their destruction while sparing healthy cells. This targeted killing of tumor cells leads to the release of potent signals, including damage-associated molecular patterns, tumor-specific antigens, and pathogen-associated molecular patterns. These signals stimulate robust innate and adaptive immune responses against the tumor. In addition to their selective tumor-killing properties and the activation of antitumor immunity, oncolytic viruses can be engineered to express additional genes, such as cytokines and chemokines, significantly boosting their effectiveness against tumors. In both immunodeficient and immunocompetent orthotopic glioblastoma (GBM) mouse models, B7-H3-targeted CAR-T cells alone were unable to inhibit GBM growth. However, when combined with the intratumor administration of CXCL11-armed oncolytic-adenovirus, they achieved a sustained antitumor response [54].

#### 3.6.4. Immune Checkpoints Inhibitors

The immunosuppressive milieu found in solid tumors induces the upregulation of intrinsic inhibitory pathways, characterized by increased expression of inhibitory receptors in T cells when interacting with their corresponding ligands within the tumor microenvironment, such as CTLA-4 and PD-1. The use of combination immunotherapy, e.g., CAR-T cell and immune checkpoint blockade could help overcome this setback. CAR-T cells engineered to secrete checkpoint inhibitors targeting PD-1 (CAR.aPD1-T) demonstrated superior functionality, expansibility, and effectiveness in eradicating tumors compared to parental CAR-T cells in a human lung carcinoma xenograft mouse model [55]. Blockade of PD-L1 in M2 TAMs in alongside CAR-T cell therapy altered phenotypes to more M1-like subsets and triggered the depletion of CD163+ M2 macrophages through interferon-γ signaling, resulting in enhanced antitumor activity of CAR-T cells [56]. A phase II study on patients with malignant pleural disease (NCT02414269) treated with autologous MSLN-CAR-T cells in combination with pembrolizumab is currently active. Among 18 patients, the median overall survival rate from CAR T-cell infusion was 23.9 months, with a 1-year overall survival rate of 83%. Stable disease persisted for ≥6 months in eight patients and two showed a complete metabolic response on a PET scan [25].

There are other ongoing clinical trials based on these approaches, including trials involving: anti-mesothelin CAR-T cells, secreting PD-1 nanobodies (NCT05373147); and anti-EGFR CAR-T cells, engineered to express the immune checkpoint antibodies anti-CTLA-4 and PD-1 (NCT03182816).

### 3.7. On-Target Off-Tumor Toxicity

Unlike hematological tumors derived from dispensable cells, targeting solid tumors requires the antigen to be a neoantigen to ensure there is no expression on healthy tissue. Neoantigens could arise from tumor-specific non-synonymous mutations, insertions, or deletions which modify the amino acid sequence of cell-surface proteins, as well as from aberrant expression of oncofetal antigens or tumor-specific post-translational modifications. Nevertheless, cell-surface neoantigens are scarce, particularly in tumors exhibiting a low mutational burden. Most CAR-T cell therapy targets for solid tumors are tumor-associated antigens (TAAs) that are also expressed on non-malignant tissues. Examples of TAAs include epidermal growth factor receptor (EGFR), HER2, carbonic anhydrase IX (CAIX), B7-H3, MSLN and the ganglioside GD2. EGFRvIII is one of the few neoantigens identified and is present in 24–67% of glioblastomas [57].

To avoid on-target off-tumor toxicity, new approaches are emerging:

Fine-tuning CAR domains. The ScFv of the CAR can be modified through mutagenesis of an existing single-chain variable fragment (scFv) or by screening scFv libraries to discover an alternative binder with varied affinity. High-affinity CAR-T cells may enhance reactivity against tumor cells with low antigen expression density, but they may also result in the recognition of target antigens found on non-tumor tissues [57].

Dual- targeting CARs. CAR-T cells targeting GPC3 and EGFR (CARgpc3-egfr) have been developed and demonstrated comparable proliferative capacity and cytotoxicity to CARgpc3 T cells against GPC3 + EGFR+ hepatocellular carcinoma (HCC) in vitro. CARgpc3 and CARgpc3-egfr T cells exhibited heightened cytokine secretion in comparison to CARegfr and mock CAR-T cells in vitro. Tumor growth suppression in vivo was better for CARgpc3-egfr T cells than for CARgpc3 T cells in GPC3 + EGFR + HCC, while such an effect was not observed for CARegfr or mock CAR-T cells [58].

## 4. CAR-T Cell Targets in Solid Tumors

A major limitation in the development of CAR-T therapies for solid tumors is the lack of targets with restricted expression on the surface of tumor cells. However, there are some promising pre-clinical developments and clinical trials of CAR-T cell therapy for solid tumors targeting various cell surface antigens.

Clinical trials currently registered for the treatment of solid tumors on clinicaltrials.gov (accessed on 16 April 2024) are listed on Table 1. We will discuss some of the most targeted antigens on CAR-T cell therapy for solid tumors below, including current approaches against carbohydrates, which hold great potential as antitumor targets in CAR-T cell therapy for solid tumors.

### 4.1. Protein Antigens

#### 4.1.1. HER2

The receptor tyrosine kinase erbB-2, commonly referred to as HER2, belongs to the EGFR family. Its overexpression is associated with the activation of signaling pathways implicated in proliferation and tumorigenesis [59]. HER2 is mainly recognized for its role in breast cancer, where HER2-targeted therapies have already been developed and approved. However, HER2 is also overexpressed in many other cancer types such as colorectal or lung cancer [60] Consequently, new strategies targeting HER2-positive cancers are under development, including CAR-T cell therapies. Currently, there are five phase I clinical trials evaluating HER-2 CAR-T cells. An interesting approach combines the administration of CAR-T cells with the intratumoral injection of an oncolytic adenovirus. The infection of cancer cells with the virus is expected to promote and facilitate CAR-T cell activation and tumor-cell killing (NCT03740256). Another notable trial is investigating the local administration of memory-enriched HER2 CAR-T cells for the treatment of brain and/or leptomeningeal metastases from HER2-positive cancers (NCT03696030). Moreover, as previously mentioned, a phase I clinical trial employing anti-HER2 CAR-M is also under development (NCT04660929).

#### 4.1.2. EGFR

Epidermal growth factor receptor (EGFR) is a cell surface receptor commonly found in certain types of cancer, such as brain or lung cancers, often in mutant hyper-active forms [61]. CAR-T cells have been developed against EGFR (EGF-CAR-T) using the piggyBac system, and they showed a potent antitumor activity against non-small-cell lung cancer (NSCLC) cell lines in vitro and in vivo in NSG mice [62]. Currently, there are three phase I and three phase I/II clinical trials evaluating anti-EGFR CAR-T cells. A notable approach uses CRISPR/Cas9 technology to knockout TGF-β receptor II, one of the major regulatory factors of the tumor microenvironment (NCT04976218). Bi-specific EGFR/B7-H3 CAR-T cells are also under study (NCT05341492), as well as EGFR/CD19 dual CAR-T cells (NCT03618381), based on the hypothesis that CD19+ B cells, functioning in their normal role as antigen-presenting cells to T cells, will enhance the expansion and persistence of the CAR-T cells. The three phase I/II clinical trials use EGFR CAR-T cells expressing antibodies anti-PD-L1/CTLA-4, underscoring the utility of checkpoint inhibition in cancer immunotherapy (NCT03182816, NCT02873390, NCT02862028).

#### 4.1.3. CEA

Carcinoembryonic antigen (CEA) is a glycoprotein implicated in cell adhesion and expressed in fetal development but absent in adult healthy tissues [63]. However, it is expressed in over 90% of colon cancers and approximately 50% of breast cancers [28]. With six phase I and four phase I/II clinical trials for CEA-CAR-T cells, it stands out as one of the most targeted antigens for CAR-T cell therapy in solid tumors. Interestingly, many of these clinical trials are exploring the intraperitoneal administration route of CAR-T cells. One trial is exploring the use of logic-gated CEA CAR-T cells inhibited by HLA-A*02, thus requiring the tumor to express CEA and have somatic loss of HLA-A*02 (NCT05736731).

#### 4.1.4. ROR1

Receptor tyrosine kinase-like orphan receptor 1 (ROR1) is a membrane-bound receptor that plays a significant role in developmental processes, with high expression levels during embryonic stages. However, its expression is typically lost or significantly reduced in adult tissues [64]. Interestingly, ROR1 is overexpressed in various human cancers, including both hematological malignancies and solid tumors, making it a potential target for immunotherapy [65]. There are five phase I clinical trials evaluating ROR1-CAR-T cells in solid tumors, of which one has been terminated due to slow accruals. A notable approach under development involves the engineering of CAR-T cells with a single non-viral transposon plasmid to express the CAR, membrane-bound IL-15 (mbIL15), a kill switch and a mechanism that blocks PD-1 expression. These engineered CAR-T cells are termed UltraCAR-T cells (NCT05694364). The incorporation of mbIL15 is aimed at enhancing the persistence and effectiveness of CAR-T cells within the tumor microenvironment, whereas the PD-1 blockade mechanism aims to counteract immune evasion mechanisms employed by tumor cells.

#### 4.1.5. NKG2D Ligands

NKG2D ligands (NKG2DLs) are membrane glycoproteins recognized by the immunoreceptor NKG2D, expressed by effector T cells and NK cells [66]. The expression of NKG2DLs is tightly controlled and inducible in response to various cellular stresses, including malignant transformation [67]. Recognition of NKG2DLs on the surface of stressed cells by the immune system leads to the elimination of the targeted cell [68]. Therefore, the NKG2D-NKG2DL axis has emerged as a novel strategy to exploit in cancer immunotherapy. Currently, there are eight phase I clinical trials exploring the use of NKG2D-based CAR-T cells. In contrast to the approved CAR-T therapies to date, which express a scFv specific for an antigen, NKG2D-based CAR-T cells express the ectodomain of NKG2D, including its transmembrane fragment. Most of them are standard CAR-T cells, but γδ CAR-T cells (NCT04107142, NCT05302037) and dual-targeting NKG2D-NKp44 CAR-NK cell alternatives are also being tested (NCT05976906). Moreover, in addition to autologous therapies, allogeneic and universal therapies are being investigated in the cited clinical trials.

#### 4.1.6. B7-H3

B7-H3, also known as CD276, is an immune checkpoint protein that is overexpressed in various types of cancers, including breast, lung, and ovarian cancers. Its limited and low expression in normal tissues makes it a potential target for cancer therapy [69]. B7-H3 plays a significant role in tumor pathogenesis by influencing various aspects such of cancer cells, such as proliferation, metabolism, and drug resistance [70]. Several phase I clinical trials are underway to explore the use of anti-B7-H3 CAR-T cells in solid tumors. One notable approach involves the use of dual anti-EGFR/B7-H3 CAR-T cells (NCT05341492). Another trial is comparing B7-H3 CAR-T cells alone with bi-specific B7-H3/CD19 CAR-T cells with/without pembrolizumab (NCT04483778).

#### 4.1.7. CD70

CD70 is a transmembrane glycoprotein of the TNF superfamily, which acts as a co-stimulatory molecule recognized by CD27 [71]. It is highly expressed in hematological and solid tumors, while being absent in most healthy tissues [72]. Currently, there are four phase I and three Phase I/II clinical trials evaluating anti-CD70 CAR-T cells in various solid tumors such as renal cell carcinoma or ovarian cancer. Similar to NKG2D-based CAR-T cells, CD70 CAR-T cells are engineered with the CD27 recognition domain.

#### 4.1.8. CLDN18.2

Claudins are a family of proteins located in the tight junctions. Claudin18.2 (CLDN18.2) expression in adult tissues is limited to gastric mucosal epithelial cells, playing a crucial role in maintaining the barrier function of the gastric mucosa [73]. Its expression is upregulated in various types of tumors, particularly those affecting the gastrointestinal tract, making CLDN18.2 a popular target for immunotherapy [74]. Currently, there are 13 phase I clinical trials and one phase I/II clinical trial assessing anti-CLDN18.2 CAR-T therapies in solid tumors. Many strategies are being tested, including dual targeting of CLDN18.2 and PD-L1/NKG2DL, or the incorporation of additional modifications to downregulate the receptor PD-1 or to express cytokines. In the phase I/II clinical trial NCT04581473, CLDN18.2 CAR-T cells are being compared with anti-PD-1 monoclonal antibodies and other chemotherapies in pancreatic cancer and advanced gastric/gastroesophageal adenocarcinoma.

#### 4.1.9. Mesothelin

Mesothelin (MSLN) is a glycosylated phosphatidylinositol-anchored protein predominantly expressed on the surface of mesothelial cells in diverse anatomical locations, including the peritoneum, pleura, pericardium, and tunica vaginalis in men [75]. It has emerged as one of the primary targets for CAR-T cell therapy in solid tumors due to its overexpression in a wide range of malignancies, including malignant mesothelioma, gastric cancer, breast cancer, ovarian cancer, pancreatic cancer, lung cancer, cervical cancer, uterine serous cancer, and cholangiocarcinoma [76]

There are currently 20 clinical trials assessing the use of MSLN CAR-T cells, making it the most targeted antigen in solid tumors. Seven of these trials are in phase I/II and different approaches are being tested. One such approach involves the expression of CD40L, a molecule implicated in the activation of the immune system [77] (NCT05693844). Additionally, combination with immune checkpoint blockade is another strategy frequently employed alongside MSLN-CAR-T treatment. CAR-T cells expressing the antibodies anti- CTLA-4 and/or PD-1 are being tested in several clinical trials. Similarly to CEA, logic-gated CAR-T cells inhibited by HLA-A*02 are also under development.

### 4.2. Carbohydrate Antigens

A notable characteristic of malignant cells is the aberrant expression of cell-surface carbohydrates, although it may not receive as much attention as other well-established hallmarks of cancer [78]. These carbohydrates that differ between healthy and malignant cells in type, structure, and/or number are known as tumor-associated carbohydrates antigens (TACAs) [79]. TACAs normally appear in the form of glycoconjugates and can be classified into proteoglycans/glycoprotein antigens and glycolipid antigens. Protein glycosylation is the most widespread protein modification observed across all cell types [80]. Cells feature a diverse range of intracellular and cell-surface-bound oligosaccharides attached to proteins and lipids. These glycoproteins and glycolipids play vital roles in extracellular processes, but they are also abundant in the cytoplasm and the nucleus, where they act as a key regulator of intracellular events. Therefore, glycoconjugates have been directly associated with biological processes such as metabolism, differentiation, or immunity [81].

The aberrant glycosylation pattern observed in TACAs contributes to multiple aspects of cancer biology, including tumor initiation, growth, invasion, metastasis, and immune evasion [82,83]. There are many mechanisms that can lead to altered protein glycosylation, including genetic mutations of enzymes involved in the synthesis and modification of proteoglycans or related proteins such as chaperones, mislocalization of these enzymes, or lack of protein substrates or nucleotide sugars and 3-phosphoadenosine-5-phosphosulfate (PAPS) [84]. Aberrant glycosylation includes alterations such as fucosylation, sialylation, O-glycan truncation, and N- and O-linked glycan branching. Malignant transformation can be accompanied by both abnormal expression of carbohydrates and the acquisition of aberrant glycosylation profiles [85]. Abnormally truncated O-glycans are also frequently found on solid tumor cells and protect cancer cells from apoptosis, promoting invasion and metastasis. Two examples include the attachment of GalNAc to Ser/Thr residues (known as Tn or CD175) and its sialylated variant, sialyl Tn (STn or CD175s). These aberrant glycan structures are associated with malignant transformation in pancreatic cancer and poor prognosis in patients with gastric cancer [86,87].

Carbohydrate antigens often exhibit low immunogenicity [88] and typically elicit T-independent immune responses [89], posing an obstacle to T-cell engagement. Despite these limitations, the implication of TACAs in cancer biology has recently sparked significant interest in their potential as targets for immunotherapy. A compelling illustration of the efficacy of carbohydrate-directed immunotherapy is the use of anti-disialoganglioside (GD2) monoclonal antibodies in combination with IL-2, GM-CSF, and isotretinoin. This therapeutic regimen has received approval from both the FDA and the EMA for treating children with high-risk neuroblastoma, resulting in notable increases in their survival rates [90]. This highlights the usefulness of carbohydrate antigens as targets for immunotherapy. Here, we will review the main TACAs involved in cancer biology, focusing on those currently under investigation as targets for CAR-T cell therapy against solid tumors in clinical trials.

#### 4.2.1. Heparan Sulfate Proteoglycans (HSPGs)

HSPGs are present on the ECM and the surface of many cellular types. Nevertheless, they have gained interest in cancer therapy due to their higher abundance on tumor cells compared to healthy ones, and their involvement in malignant transformation [91]. HSPGs play a wide variety of roles including cell adhesion and migration, maintenance of tissue integrity, and signal transduction, as well as regulation of cell growth and proliferation. HSPGs have been shown to enhance fibroblast growth factor receptor signaling, thereby promoting the growth of different types of tumors [92]. Heparan sulfate can be found linked to different cell surface proteins, but it is mainly present on two membrane-anchored proteoglycans: glypicans and syndecans [93].

Glypicans are cell-surface HSPGs directly linked to membrane phospholipid phosphatidylinositol (GPI) [94]. Depending on the core protein bound to the HS chain, and the number of chains present, six different families have been described: GPC1 to GPC6. Glypicans can regulate the Wnt signaling pathway, modulating cell growth, and GPC1, GPC2, and GPC3 are overexpressed in a several tumors. GPC1 was found to be overexpressed in human pancreatic cancer compared to healthy pancreatic cells, and it is associated with poor patient prognosis [95]. High levels of this proteoglycan have also been described in breast cancer [96] and glioma; it is a marker of poor prognosis in glioblastoma [97]. GPC2 is highly expressed in neuroblastoma, and not detectable in healthy cells. Moreover, its presence is associated with poorer prognosis and overall survival [98]. Expression of GPC3 has been found in hepatocellular carcinoma by several authors; it was found to be absent or expressed at a low level in healthy liver or benign liver diseases [99]. Furthermore, overexpression of GPC3 has been found in urothelial carcinoma, thyroid cancer, ovarian clear cell carcinoma, melanoma, lung squamous cell carcinoma, and salivary gland tumors [97]. In pancreatic and colorectal cancer, the expression of GPC4 is increased [100,101] and higher levels of GPC6 mRNA have been described in gastric cancer compared to healthy tissue [102]. However, a decrease in certain glypicans, such as GPC4, whose expression is downregulated in metastatic breast cancer compared to non-metastatic tumors, has also been linked to tumor progression [103], and downregulation of GPC5 has been observed in non-small-cell lung cancer [104] and breast tumors [105].

Due to the tumor-specificity of glypicans, these HSPGs constitute a promising target for tumor therapy. Vaccination with GPC3 peptides has demonstrated safe and specific immune responses, and prolonged recurrence-free survival in patients with hepatocellular carcinoma in a phase I and II clinical trial [106,107]. Several therapeutic monoclonal antibodies targeting GPC3 have been developed, such as GC33, which has demonstrated antibody-dependent cell-mediated cytotoxicity against GPC3-positive hepatocellular carcinoma (HCC) cell lines and inhibition of tumor growth in mouse models of HCC [108]. In a phase I trial, patients with advanced HCC showed good tolerability towards GC33, and some patients with high GPC3 expression showed signs of tumor reduction [109].

CAR-T cell therapy directed against glypicans has also been developed. Anti-GPC1 human and murine CAR T-cells demonstrated antitumor effects in xenogeneic and syngeneic solid-tumor mouse models, with no obvious adverse effects [110]. In a neuroblastoma mouse model, anti-GPC2 CAR-T cells showed potent antitumor in vivo efficacy [98]. Regarding GPC3, there are six clinical trials investigating CAR-T cell therapies targeting this antigen. A novel and unique phase I/II clinical trial (NCT05120271) is utilizing CAR-T cell therapy directed against GPC3 that co-expresses exogenous glutamic-oxaloacetic transaminase 2 (GOT2), an enzyme involved in glutamine metabolism crucial for proper T cell function [111]. These CAR-T cells, named BOXR1030, have shown superior effectiveness in aggressive solid-tumor xenograft models in vivo. They also exhibit enhanced cytokine production profile, a less-differentiated T cell phenotype characterized by reduced expression of stress and exhaustion markers, and increased proliferation in conditions mimicking the TME in vitro [112].

Syndecans represent the other major group of HSPGs. They are membrane-bound proteins consisting of a highly conserved C-terminal cytoplasmic region, a transmembrane domain, and an extra-cellular N-terminal domain linked to multiple glycosaminoglycan chains of heparan-sulfate or chondroitin-sulfate [113]. The difference between these two glycosaminoglycans are that heparan-sulfate consists of multiple disaccharide units of glucuronic acid linked to N-acetylglucosamine [114], and chondroitin-sulfates are unbranched polysaccharides of D-glucuronic acid and N-acetyl-D-galactosamine. Both glycosaminoglycans are found sulfated in varying amounts [115].

Syndecans’ expression has been found to become dysregulated in tumorigenesis. Syndecan-1 was first identified as a transmembrane protein that bound ECM components to epithelial cells [93], and its expression is upregulated in breast cancer [116] and ovarian cancer [117]. Furthermore, higher serum levels of this HSPG have been found in primary colorectal cancer [118], multiple myeloma [119] and small- and non-small-cell lung cancer [120]. In colorectal cancer, higher expression of syndecan-2 [121] is described, as well as in lung adenocarcinoma [122] and pancreatic tumors [123], where syndecan-3 is also elevated [124]. Syndecan-4 expression is upregulated in grade 4 glioblastoma [125] and osteosarcoma [126].

A CAR-T for the treatment of multiple myeloma against syndecan-1 (CD138) has been developed. Five patients with chemotherapy-refractory multiple myeloma were treated with CART-138 cells, causing a stabilization of the disease in four patients and a reduction of the tumor myeloma cells in one [127]. There is an ongoing phase I clinical trial to test the safety of CAR138 T cells infusion in multiple myeloma patients (NCT03672318).

#### 4.2.2. Mucin Glycans

Mucins are high-molecular-weight and heavily glycosylated proteins (glycoproteins) that are the main component of the mucus. They are produced by goblet cells in the respiratory, gastrointestinal, and urogenital tracts, where they form a protective barrier against external agents and provide lubrication to the epithelial barrier, contributing significantly to the overall health and function of these mucosal surfaces [128]. Mucins can be subclassified into membrane-bound (13 genes), secreted (six genes), and atypical mucins (two genes) [129]. The main types of glycosylation found in the structure of mucins are O-glycosylation and N-glycosylation, both of which are key factors in the biological properties and functions of these glycans [130,131]

MUC1, a membrane-bound mucin, is the most studied tumor mucin, as it is closely associated with the development of cancers of glandular epithelial origin. Its overexpression, loss of cell surface polarization, infraglycosylation, and/or aberrant glycosylation is a common finding in multiple adenocarcinomas such as lung, liver, colon, breast, pancreatic, and ovarian cancer [132]. There are six ongoing phase I/II clinical trials studying MUC1 as a target for CAR-T cells. Most of them combine CAR-T treatment with immune checkpoint blockade, by PD-1 knockout for the treatment of patients with advanced MUC1-positive breast cancer (NCT05812326). Other clinical trials combine MUC1-CAR-T cells with the expression of immune checkpoint antibodies (CTLA-4 and PD-1) for the treatment of adult patients with MUC1-positive, advanced recurrent or refractory malignant solid tumors (NCT03179007). In a preclinical model of head and neck squamous cell carcinoma (HNSCC), MUC1-CAR-T cells specifically killed MUC1+ HNSCC cell lines in vitro and secreted IL-2, interferon γ (IFN-γ), and TNF-α. To improve T cell function, a fourth generation CAR-T cell that could secrete IL-22 was generated, and it showed improved cytotoxic capacity in vitro and in vivo [133].

The most frequent aberrant glycosylation found in MUC1 are the Tn antigen and the STn antigen. These altered glycoproteins have been associated with reduced sensitivity to chemotherapy and exhibit immune inhibitory properties in breast cancer [134]. The almost exclusive presence of these variations on cancer cells has made them suitable targets for CAR-T cell therapy and can decrease on-target off-tumor toxicity. CAR-T cells targeting the Tn and STn antigen on MUC1 have shown specific recognition on different tumor cells in vitro and displayed effective antitumor activity in murine models of cancers, including leukemia and pancreatic cancer [135].

#### 4.2.3. Gangliosides

Gangliosides are glycosphingolipids bound to the cell membrane and mostly expressed in neural tissue. However, specific gangliosides are overexpressed in certain tumors. The promotion of tumorigenesis by disialogangliosides (or complex gangliosides) has been described and, although they play fundamental roles in developmental stages, they reduce or lose their expression in non-neural healthy adult cells [136]. Mono-sialylgangliosides, on the other hand, are thought to suppress tumor-cell phenotypes [137]. GD2 and GD3 are di-sialogangliosides overexpressed in melanoma [138], sarcoma [139], glioblastoma [140], neuroblastoma [141], breast cancer [142], pediatric T cell lymphomas [143], and lung cancer [144].

Anti-GD2 monoclonal antibodies have had a great impact in cancer immunotherapy against neuroblastoma [90]. Ganglioside GD2 is a glycosphingolipid linked to sialic acid residues that is abundantly expressed on cancers of neuroectodermal origin, including neuroblastoma and melanoma [85]. Currently, there are one phase I and four phase I/II clinical studying anti-GD2 CAR-T cells. A phase I clinical trial using OX40/CD28 expressing anti-GD2 CAR-T cells has been completed but no results have been posted to date (NCT02107963). In an academic phase I/II trial (NCT03373097), an anti-GD2 CAR-T expressing the inducible caspase 9 suicide gene (GD2-CART01) was studied for the treatment of patients with high-risk neuroblastoma. In 20 of 27 patients (74%), cytokine release syndrome occurred, and it was mild in 19 of 20 cases (95%). In one patient, the suicide gene was activated, resulting in the elimination of GD2 CART01. GD2-targeted CAR-T cells proliferated in vivo and were detectable in peripheral blood in 26 of 27 patients up to 30 months after infusion, with a median persistence of 3 months, ranging from 1 to 30 months. In total, 17 children exhibited a response to the treatment (overall response, 63%). Among them, nine patients achieved a complete response, while eight had a partial response [145]. Another novel strategy under development consists of the expression of the IL-7 receptor C7R (NCT03635632). The co-expression of this receptor together with the CAR has shown increased antitumor activity in preclinical models [146].

#### 4.2.4. Blood-Group Related Lewis Antigens

Lewis blood group antigens are specific carbohydrate structures found on the surface of red blood cells. These antigens are formed by different arrangements of fucose and N-acetylglucosamine and can be found conjugated with proteins or with lipids. Lewis antigens appear in red blood cells by adsorption to Lewis glycolipids of serum [147]. Blood-group-related TACAs include Lewis Y, Lewis X, Lewis A, and their sialylated forms. Most of them have been found to be aberrantly expressed in several tumor cells [148,149], but in the context of CAR-T cell therapy against solid tumors, Lewis Y (LeY) has received the most attention.

LeY is a blood-group-related molecule comprising di-fucosylated carbohydrate residues attached to various proteins and lipids, including CA-125 and MUC1 [150]. Many cancers express high levels of LeY, including lung, colorectal, breast, ovarian, and prostate cancers. However, its expression is low or limited in healthy tissues [151]. A phase I clinical trial has been carried out to evaluate the safety and tolerability of LeY CAR-T cells in patients with Lewis Y antigen-expressing, advanced solid tumors (NCT03851146). In another phase I trial investigating autologous chimeric antigen receptor (CAR) anti-LeY T-cell therapy for acute myeloid leukemia (AML), no safety concerns were reported. Among the patients, one attained cytogenetic remission, another experienced a reduction in peripheral blood blasts despite ongoing leukemia activity, and a third sustained a prolonged remission. Furthermore, the infused CAR -T cells were detectable for up to 10 months following administration [152].

## 5. Conclusions

CAR-T cell therapy has proven efficacy for the treatment of hematological malignancies, such as B cell leukemias and lymphomas, and multiple myeloma. Nevertheless, it has not achieved similar success in the treatment of solid tumors, despite the number of preclinical developments and ongoing clinical trials. The hostile tumor microenvironment and the increased difficulty for CAR-T cells to infiltrate solid tumors may be responsible for the lack of phase III trials and approved therapies. However, the large number of experimental approaches to circumvent these difficulties, including many studies that are underway, some already in clinical trials, suggest a bright future for CAR-T therapy for solid tumors. Given the limitations of target antigens, tumor-associated carbohydrates are increasingly being explored as optimal targets for this therapy. These carbohydrates can be highly tumor-specific, while being shared among tumors from different individuals and different tissues of origin; therefore, CAR-T cell therapy may have a promising “sweeter” future.

## Figures and Tables

**Table 1:** Current ongoing clinical trials.

Protein Antigens			
Antigen	Total	CAR-T Interventions	Phase
Mesothelin (MSLN)	20	Anti-MSLN CAR-T cells, anti-CTLA4/PD-1 expressing MSLN CAR-T cells, CD40L expressing MSLN CAR-T cells, logic-gated MSLN CAR-T cells inhibited by HLA-A*02	I/II
HER2	6	Anti-HER2 CAR-T cells, oncolytic viruses + CAR-T cells, memory-enriched anti-HER2 CAR-T cells	I
CEA	10	Anti-CEA CAR-T cells, logic-gated CEA CAR-T cells inhibited by HLA-A*02	I/II
ROR1	5	Anti-ROR1 CAR-T cells, anti-ROR1 CAR-T cells expressing mbIL15 and PD-1 downregulation	I/II
B7-H3	7	Anti-B7H3 CAR-T cells, dual anti-EGFR/B7H3 CAR-T cells, dual anti-CD19/B7H3 CAR-T cells with/without pembrolizumab	I
BT-001	2	Anti BT-001 CAR-T cells	I
CD70	7	Anti-CD70 CAR-T cells	I/II
NKG2DL	8	Anti-NKG2DL CAR-T cells, dual anti-NKG2DL/CLDN18.2 CAR-T cells, allogeneic/haploidentical anti-NKG2DL CAR γδ T cells, universal dual anti-NKG2DL-NKp44 CAR-T cells	I
EGFR	6	Dual anti-EGFR/B7H3 CAR-T cells, anti-CTLA-4/PD-1 expressing EGFR CAR-T cells, TGFβR knockout anti-EGFR CAR-T cells, dual anti-EGFR/CD19 CAR-T cells	I/II
CLDN18.2	14	Anti-CLDN18.2 CAR-T cells, dual anti-CLDN18.2/PD-L1 CAR-T cells, dual anti-NKG2DL/CLDN18.2 CAR-T cells, anti-CLDN18.2 CAR-T cells with co-expression of cytokines, iPD-1-CLDN18.2 CAR-T cells	I/II
PD-L1	2	dual anti-CLDN18.2/PD-L1 CAR-T cells, dual anti-VEGFR1/PD-L1 CAR-T cells	I
VEGFR1	1	dual anti-VEGFR1/PD-L1 CAR-T cells	I
CD22	1	Anti-PD-L1 armored anti-CD22 CAR-T/CAR-TILs	I
EpCAM	2	Anti-EpCAM CAR-T cells	I
TM4SF1	1	Anti-TM4SF1 CAR-T cells	N/A
Nectin4	1	Anti-Nectin4/FAP CAR-T cells expressing IL-7 and CCL19 or/and IL-12	I
CLDN6	1	Anti-CLDN6 CAR-T cells with or without a liposomal formulation containing CLDN6 RNA	I/II
PSMA	1	Dual anti-GD2/PSMA CAR-T cells	I/II
ROR2	1	Anti-ROR2 CAR-T cells	I
EphA-2	2	Anti-EphA-2 CAR-DCs loaded with TP53/KRAS mutant peptide combine with anti-PD-1/CTLA4 antibodies	I
HLA-G	2	Anti-HLA-G CAR-T cells, anti-HLA-G-BiTE γδ CAR-T cells,	I/II
GCC	2	Anti-GCC CAR-T cells	N/A
**Carbohydrate antigens**			
**Antigen**	**Total**	**CAR-T interventions**	**Phase**
MUC1	7	Anti-MUC1 CAR-T cells, anti-CTLA-4/PD-1 expressing MUC1 CAR-T cells, allogeneic anti-MUC1 CAR-T cells, PD-1 knockout anti-MUC1 CAR-T cells	I/II
GPC3	6	Anti-GPC3 CAR-T cells, IL-15 armored anti-GPC3 CAR-T cells, IL-15 and IL-21 armored anti-GPC3 CAR-T cells, anti-GPC3 CAR-T cells with exogenous GOT2 expression	I/II
Lewis Y	1	Anti-Lewis Y CAR-T cells	I
GD2	6	Anti-GD2 CAR-T cells, Dual anti-GD2/PSMA CAR-T cells, anti-GD2 CAR-T cells with an inducible apoptotic caspase 9 domain (iC9), OX40/CD28 expressing iC9 anti-GD2 CAR-T cells, C7R expressing anti-GD2 CAR-T Cells	I/II
Trop-2	1	Anti-Trop-2 CAR-T cells	I/II
CD138	1	Anti-CD138 CAR-T cells	I
